# High vs. low vancomycin therapeutic concentrations in periprosthetic joint infection: A retrospective cohort analysis

**DOI:** 10.3389/fphar.2025.1555276

**Published:** 2025-03-27

**Authors:** Jingdong Cheng, Dehua Wang, Yanqing Chen, Qingqing Zhao, Qianyi Ou, Liangming Zhang, Xinyu Li

**Affiliations:** ^1^ Department of Pharmacy, The First Affiliated Hospital of Chongqing Medical University, Chongqing, China; ^2^ Department of Pharmacy, Panzhihua Central Hospital, Panzhihua, Sichuan, China; ^3^ Department of Orthopedics, The First Affiliated Hospital of Chongqing Medical University, Chongqing, China

**Keywords:** vancomycin, therapeutic drug monitoring, periprosthetic joint infection, treatment effectiveness, treatment safety

## Abstract

**Objective:**

Current guidelines recommend vancomycin concentrations of 10–20 μg/mL for most infections, with higher levels (15–20 μg/mL) suggested for severe cases. However, evidence supporting these recommendations in periprosthetic joint infection (PJI) is limited. This study aims to evaluate the impact of different vancomycin concentration ranges (10–15 vs. 15–20 μg/mL) on the safety and effectiveness in PJI population.

**Methods:**

This retrospective study included 37 patients with vancomycin Therapeutic Drug Monitoring due to periprosthetic joint infection. Patients were categorized into two groups according to vancomycin concentrations, low concentration group (10–15 μg/mL) and high concentration group (15–20 μg/mL). Patients were followed up for at least 2 years. The long term clinical outcomes, inflammatory markers, as well as adverse events were compared. A physiologically based pharmacokinetic model was established to compare vancomycin distribution in kidney and bone marrow between the two groups.

**Results:**

There were 23 (62.16%) patients classified as the HC group and 14 (37.84%) as the LC group. The average steady-state trough concentration (Css) in the HC group was 17.74 μg/mL, and in the LC group was 12.11 μg/mL. At the end of follow-up, two patients (5.40%) in the HC group had died, and one (2.7%) was readmitted for joint fusion due to recurrent infections, whereas no deaths or readmissions occurred in the LC group. However, no significant differences were identified. Similar improvements from baseline were observed across WOMAC, Harris, HSS, and SF-12 scores between the groups. The synovial white blood cell (WBC) count was significantly lower in the HC group compared to the LC group (5,481 vs. 7,106/μL, *P* = 0.009), with a more pronounced reduction from baseline noted. The PBPK model showed a greater increase in drug distribution to the bone marrow in the HC group (20.66 μg/mL vs. 14.34 μg/mL), with a smaller rise in the kidney (376.2 μg/mL vs. 327.7 μg/mL).

**Conclusion:**

Maintaining vancomycin concentrations of 15–20 μg/mL is associated with better infection control for PJI patients who present with higher synovial WBC account, without compromising patient safety, joint function, or long-term quality of life.

## 1 Introduction

Vancomycin is one of the most widely used antibiotics for the management of serious Gram-positive bacterial infections. It is also among the first line treatment for infections caused by Methicillin-Resistant *Staphylococcus aureus* (MRSA) (Hsu et al., 2018; Chanapiwat et al., 2023). Because of its narrow therapeutic range, vancomycin therapy is associated with many serious adverse reactions such as acute kidney injury (AKI) and ototoxicity (Woldu and Guglielmo, 2018). Thus, it is crucial to monitor the serum concentration of vancomycin to maximize effectiveness while minimizing these potential adverse effects (Al-Maqbali et al., 2022). Extensive pharmacokinetic studies have been conducted across various patient populations such as pediatric (Al-Mazraawy and Girotto, 2021; Moriyama et al., 2021) and intensive care unit (ICU) patients (Chuma et al., 2018; Hou et al., 2021; Viertel et al., 2023), and in individuals with severe with severe Gram-positive bacterial infections (Prabaker et al., 2012). Drug monitoring has enabled clinicians to target vancomycin levels within a narrow range, enhancing both safety and effectiveness.

PJI is a serious and challenging complication following total joint arthroplasty, associated with high morbidity, substantial economic, psychological burdens due to frequent readmissions and repeated surgical interventions (Leta et al., 2024; Zardi and Franceschi, 2020; Schwarz et al., 2019; Patel, 2023; Belden et al., 2019; Zhang et al., 2020). *Staphylococcus aureus*, *Staphylococcus* epidermidis, and other coagulase-negative staphylococci are the primary pathogens implicated in PJI (Drago et al., 2019; Simon et al., 2022; Xu et al., 2022). Vancomycin is widely used to treat PJI (Yoon et al., 2017), which often involves prolonged hospital stays and extensive antibiotic therapy. Therefore, therapeutic drug monitoring of vancomycin is necessary to achieve an optimal balance between effectiveness and safety in these cases. Although the ratio of area under the curve to minimum inhibitory concentration (AUC/MIC ratio) is the preferred parameter for pharmacodynamic monitoring, trough serum vancomycin concentration continues to serve as a practical measure of effectiveness in many healthcare facilities, and has also been recommended as an option for therapeutic drug monitoring of vancomycin by some guideline including those from Chinese Pharmacological Society (He et al., 2020).

Current guidelines recommend the therapeutic serum concentration of vancomycin within 10–20 μg/mL for most infections, with 15–20 μg/mL suggested for severe cases (He et al., 2020; Rybak et al., 2020). Sustaining elevated vancomycin levels may augment its antimicrobial effectiveness; however, this practice could also elevate the risk of organ toxicity or damage (Álvarez et al., 2016). Moreover, these recommendations for the higher concentration range (15–20 μg/mL) are primarily derived from studies focused on severe infections such as bacteremia, pneumonia, and infective endocarditis, which offer limited evidence specifically related to PJI. Given that PJI predominantly affects elderly patients and often necessitates prolonged antibiotic therapy (Weinstein et al., 2023), the balance between effectiveness and safety when maintaining elevated vancomycin levels in this population remains uncertain (Bue et al., 2018). To address this gap, our study conducted a retrospective analysis of PJI patients treated with vancomycin at our hospital from 2018 to 2022. This study aims to evaluate the impact of different vancomycin serum concentration ranges (10–15 μg/mL vs. 15–20 μg/mL) influence long term clinical outcomes, inflammatory markers, and adverse events. Our findings aim to inform vancomycin dosage adjustment and monitoring in PJI patient population.

## 2 Materials and methods

### 2.1 Patients selection

#### 2.1.1 Inclusion/exclusion criteria

This retrospective study was reviewed and approved by the Ethical Committee of the hospital. Between 2018 and 2022 (Hsu et al., 2018), adult patients (≥18 years) receiving vancomycin therapy during a single hospital admission to Orthopedic Surgery Ward of the First Affiliated Hospital of Chongqing Medical University (Chanapiwat et al., 2023), with surgery record (Woldu and Guglielmo, 2018), with at least one Css record were included in this study. Patients were excluded based on the following criteria (Hsu et al., 2018): with at least one Css exceeding 20 μg/mL or falling below 10 μg/mL (Chanapiwat et al., 2023); diagnosed with infections other than PJI Musculoskeletal Infection Society (MSIS) diagnostic criteria (2018); and (Woldu and Guglielmo, 2018) presence of fungal infections or gram-negative infections.

#### 2.1.2 Grouping of patients

All patients received vancomycin treatment through intermittent infusion. The steady-state trough concentration was collected after the third or fourth dose, 30 min before the next dose, in accordance with the American Society for Health System Pharmacists guidelines (Rybak et al., 2020). We only considered adjusting the dosing when the initial vancomycin concentration was outside the therapeutic range. If two or more steady-state serum concentrations of vancomycin were reported, then the average concentration was calculated and used for analysis. Patients were divided into two groups according to steady-state vancomycin trough concentrations: patients with concentrations 10–15 μg/mL were defined as the LC group, patients with concentrations 15–20 μg/mL were defined as HC group.

### 2.2 Outcomes and covariates

The primary outcome measures of this study are as follows (Hsu et al., 2018): 2-year all-cause mortality and readmissions (Chanapiwat et al., 2023). The recurrence rate of infection in patients (Woldu and Guglielmo, 2018). The long-term survival conditions of patients include the patients’ quality of life (measured by The Western Ontario and McMaster Universities Arthritis Index (WOMAC) and SF12 scores) and hip and knee joint function scores (measured by Harris score andHospital for Special Surgery Score (HSS) scores) (Al-Maqbali et al., 2022). The occurrence of liver and renal injury during vancomycin therapy, and other adverse drug reactions (Al-Mazraawy and Girotto, 2021). The declination in inflammatory indicators, including C-reactive protein (CRP), erythrocyte sedimentation rate (ESR), serum WBC, synovial WBC count and synovial primary membranous nephropathy percent (PMN%) at the time of patient discharge.

AKI is defined as an increase in the serum creatinine (SCr) level of ≥0.3 mg/dL, or a 50% increase from baseline in consecutive daily readings, or a decrease in calculated creatinine clearance (CrCl) of 50% from baseline on two consecutive days. drug-induced liver injury (DILI) is defined as total bilirubin (TBil) >23 μmol/L or a two-fold increase in serum aspartate transaminase (AST) and alanine aminotransferase (ALT) levels. Leukopenia is defined as WBC <4.0 × 10^9^/L in patients due to various factors. Other adverse drug reactions include the occurrence of rashes, red man syndrome, tinnitus, et,al.

Mean CrCl was determined by the Cockcroft-Gault equation: Creatinine clearance = [(140 - age) × body weight (kg)]/[0.818 × creatinine (μmol/L)], and for women, the calculated result is multiplied by 0.85. The AUC of vancomycin was predicted by the Bayesian model, and the MIC value of the bacteria is determined by the results of drug susceptibility according to the Clinical and Laboratory Standards Institute breakpoints. If the bacterial culture result was negative, 1 mg/L value was used for MIC.

The selection of surgical strategies, including DAIR, one-stage revision, and two-stage revision, is determined based on patients’ clinical manifestations, infection duration, and infection type (Peng et al., 2023). DAIR is indicated for acute or localized infections in patients with well-preserved periarticular soft tissue conditions and pathogens susceptible to antibiotics. The procedure involves complete excision of infected soft tissues, joint cavity irrigation, polyethylene liner exchange with prosthesis retention, and local antibiotic administration. One-stage revision is appropriate for patients with confirmed pathogen identification, antibiotic susceptibility, and favorable soft tissue integrity. The surgical protocol entails prosthesis removal, thorough debridement, and immediate prosthetic reimplantation. Two-stage revision is reserved for chronic infections, complex cases caused by drug-resistant bacteria or unidentified pathogens, or patients with compromised soft tissue conditions. The first stage involves prosthesis removal, radical debridement, and placement of an antibiotic-loaded cement spacer combined with systemic antibiotic therapy. Prosthesis reimplantation is performed after infection eradication (typically 6–8 weeks later).

### 2.3 Data collection and organization

The patients’ data was collected from the electronic records, including the demographic data (e.g., age, sex), initial body mass index (BMI), comorbidities (such as hypertension, diabetes), duration of vancomycin therapy, average daily vancomycin dosage, length of hospital stay, vancomycin trough concentrations, microbiological data, surgical interventions (one-stage revision, two-stage revision and debridement and implant retention), the site of infection (knee or hip). Before the initiation of vancomycin therapy and at discharge, the laboratory tests results including WBC, CRP, ESR, synovial WBC count, PMN%, ALT, AST, TBil, CrCl were also documented. If two or more steady-state serum concentrations of vancomycin were reported, then the average concentration was calculated and used for analysis. patients were contacted via telephone or during outpatient follow-up visits to assess outcomes using the WOMAC score, SF-12 score, Harris Score, or HSS score.

### 2.4 Vancomycin physiologically based pharmacokinetic (PBPK) model construction, validation and prediction

To assess pharmacokinetics and tissue distribution of vancomycin in PJI patients, we employed the commercially available dynamic PBPK modeling software GastroPlus™ version 9.9 (Simulations Plus, Inc.) to construct a stepwise model as previously described (Du et al., 2020). This model was then used to predict vancomycin distribution in the kidney and bone marrow of PJI patients. The modeling and simulation process was divided into four key stages (Hsu et al., 2018): The molecular structure of vancomycin was imported into GastroPlus™ 9.9 software, where the physicochemical parameters and dosing regimen were configured (Chanapiwat et al., 2023). Using clinical trial data from the literature (Cutler et al., 1984), the physiological and anatomical parameter database for young adults in the United States was selected. A PBPK model for intravenous vancomycin administration in this population was developed and validated by comparing the predicted vancomycin concentration-time profiles with observed clinical data obtained from young adults following intravenous infusion (Woldu and Guglielmo, 2018). The PBPK model for U.S. young adults was then adapted for Chinese young adults, incorporating relevant physiological data for this population. Plasma concentration-time data reported in the literature for Chinese young adults who received a single 1.0 g intravenous infusion of vancomycin (Du et al., 2018) was used to systematically compare the model’s predictions, allowing for an evaluation of its accuracy and reliability in this cohort (Al-Maqbali et al., 2022). In the established PBPK model for the Chinese population, physiological parameters such as age, BMI, and renal function were adjusted to reflect the characteristics of HC and LC group in our study, thus generating a model specific to PJI patients. The model was then validated using Css data obtained from our PJI patient cohort. Following validation, the PBPK model was employed to predict vancomycin concentration-time profiles in kidney and bone marrow. Verification of the established PBPK model was primarily based on AUC_0-t_ and C_max_ were predicted and compared with published data. The predicted mean population PK parameters of the drug should fall within 70%–130% of the observed values.

### 2.5 Data analyses

Continuous characteristics of patients are presented using measures of the central tendency, mean of normally distributed data, or median for skewed data as assessed using Shapiro-Wilks test, and variability was estimated using variance or interquartile range accordingly. Baseline demographic and clinical characteristics between high trough and low trough patients were compared using Mann-Whitney test. Categorical variables were analysed using a chi-square or Fisher’s exact test, and continuous variables were analysed using Student’s t-test or a Mann–Whitney test for parametric or nonparametric variables, respectively. The statistical significance level was set at P < 0.05. We used inverse probability of treatment weighting (IPTW) to adjust for confounding due to differences between the groups, assigning a weight of mean of propensity scores (PS)/PS for the HC group and (1 - means of PS)/(1 - PS) for the LC group, where PS is the probability that each individual will be assigned to the older group. We computed the standardized mean difference to assess the balance of variables; we confirmed the standardized mean difference to be <0.1 for all matching variables except for age and CrCl. We analysed the differences for the categorical and continuous variables between the two groups using a weighted Wilcoxon test and a weighted t-test, respectively.

## 3 Results

### 3.1 Patients’ clinical characteristics

**FIGURE 1:**
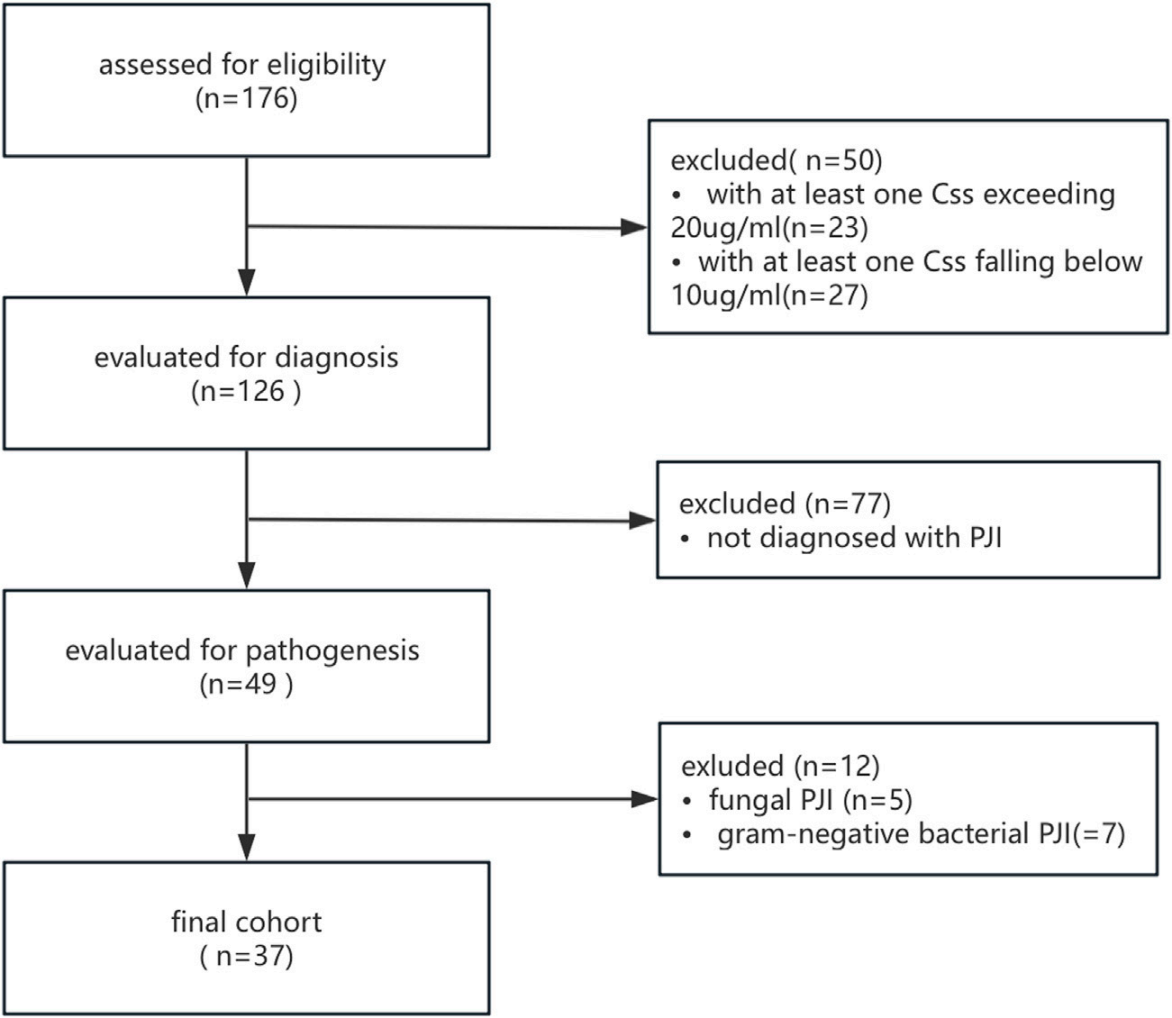
Flow chart of the selection process for study.

A total of 176 patients from othorpedic surgery ward were screened for analysis, Patients with at least one vancomycin Css outside the therapeutic window, those with non-PJI infections, and individuals with PJI caused by fungi or gram-negative bacteria were excluded (Figure 1). 37 patients were included in the final analysis, with 23 patients (62.16%) classified as the HC group and 14 patients (37.84%) classified as the LC group (Table 1). The average age of patients in the HC group was 72.61 years, significantly higher than the 54.86 years observed in the LC group (P < 0.05). No statistically significant differences were found between the two groups in terms of body mass index (BMI), gender, history of diabetes, or smoking history.

**TABLE 1:** Patients’ clinical characteristics.

Characteristics	HC group (n = 23)	LC group (n = 14)	*P* value
Age (year)	72.61 ± 12.66	54.86 ± 11.59	0.001
Man (%)	9 (39.13%)	8 (57.14%)	0.328
BMI(Kg/m^2^)	23.91 ± 3.53	25.04 ± 3.73	0.360
Hypertension	10 (43.48%)	5 (35.71%)	0.641
Cardiovascular diseases	7 (30.43%)	3 (21.43%)	0.710
COPD	5 (21.74%)	2 (14.29%)	0.575
diabetes	4 (17.39%)	1 (7.14%)	0.377
CRP (mg/L)	23.80 (8.68, 42.90)	19.85 (4.32, 36.43)	0.572
ESR (mm/h)	72.43 ± 35.25	56.07 ± 39.50	0.199
WBC (10^9^/L)	6.37 (5.24, 9.90)	6.70 (5.69, 8.99)	0.957
ALT (U/L)	21.0 (14.0, 27.0)	23 (13.25, 36.75)	0.847
AST (U/L)	19 (15.0, 28.0)	19 (14.0, 23.5)	0.614
TBil (μmol/L)	9.80 (5.50, 11.70)	7.85 (5.55, 12.33)	0.817
CrCl mL/(min×1.73 m^2^)	71.87 ± 26.69	111.1 ± 34.66	0.001
TKA (%)	12 (52.18%)	7 (50.0%)	0.999
THA (%)	11 (47.82%)	7 (50%)	0.999
Patients categories	—	—	0.999
Early PJI	9 (39.13%)	6 (42.86%)	
Delayed PJI	11 (47.83%)	6 (42.86%)	
Chronic PJI	3 (13.04%)	2 (14.28%)	
Surgical procedure (%)	—	—	0.921
DAIR	7 (30.34%)	4 (28.58%)	
One-stage revision	6 (26.09%)	5 (35.71%)	
Two-stage revision	10 (43.57%)	5 (35.71%)	
Microorganisms (%)	17 (73.9%)	5 (26.3%)	0.038*
Methicillin-sensitive *Staphylococcus epidermidis*	4 (17.39%)	0	
Methicillin-resistant *Staphylococcus epidermidis*	5 (21.74%)	2 (14.29%)	
Methicillin-sensitive *Staphylococcus aureus*	2 (8.70%)	3 (21.43%)	
Methicillin-resistant *Staphylococcus aureus*	2 (8.70%)	0	
*S.dysgalactiae equisimilis*	2 (8.70%)	0	
*streptococcus mitis*	1 (4.35%)	0	
*Enterococcus Faecium*	1 (4.35%)	0	

BMI, body mass index; COPD,chronic obstructive pulmonary disease; *: significant difference (<0.05).

AT the time admission, the average baseline CrCl of HC group was 71.87 mL/(min×1.73 m^2^), compared with 111.1 mL/(min×1.73 m^2^) of the LC group (*P* = 0.001). Baseline serum levels of TB, AST, ALT, as well as CRP, ESR, WBC were found with no significant difference. The surgical approaches were also comparable between the two groups (*P* > 0.05). The main pathogenic microorganisms identified in the HC group included *Staphylococcus* (56.52%) and *Streptococcus* (13.04%). While the types of main pathogens in the LC group were similar, the overall positive culture rate was lower (26.3% vs. 73.9%, P = 0.038).

### 3.2 Vancomycin Css and AUC/MIC

During the hospital stay, the average Css in the HC group was 17.74 μg/mL, compared to 12.11 μg/mL in the LC group, indicating a statistically significant difference (*P* < 0.05). The AUC/MIC value in the HC group was 630.2, and that in the LC group was 450.8, presenting a statistically significant difference (*P* < 0.05). The mean AUC/MIC ratio for both groups reached the effectiveness level recommended by the guidelines which is ≥400. Nevertheless, there was no statistically significant difference in the duration of vancomycin therapy and daily dosages between the two groups (Table 2). The duration of treatment for patients in the HC group ranged from 6 to 49 days, while for those in the LC group, it ranged from 6 to 46 days. The MICs of vancomycin for each strain were recorded in Table 3.

**TABLE 2:** Parameters related with vancomycin therapy.

Parameters	HC group (n = 23)	LC group (n = 14)	*P* value
The duration of vancomycin therapy (day)	18.0 (14, 26)	17.5 (10.5, 21.25)	0.177
Average daily vancomycin dosage(g)	2.0 (1.70, 2.0)	2.0 (2.0, 2.0)	0.307
Steady-state vancomycin trough concentration (mg/L)	17.74 ± 1.48	12.11 ± 1.65	0.001*
AUC/MIC	630.2 ± 216.1	450.8 ± 151.6	0.004*

*: significant difference, *P* < 0.05.

**TABLE 3:** The MICs of vancomycin for each strain.

Microorganisms (%)	HC group	LC group
Methicillin-sensitive *Staphylococcus epidermidis*	1 μg/mL (n = 4)	0
Methicillin-resistant *Staphylococcus epidermidis*	1 μg/mL (n = 4) 2 μg/mL (n = 1)	1 μg/mL (n = 2)
Methicillin-sensitive *Staphylococcus aureus*	≤0.5 μg/mL (n = 1) 1 μg/mL (n = 1)	≤0.5 μg/mL (n = 2) 1 μg/mL (n = 1)
Methicillin-resistant *Staphylococcus aureus*	≤0.5 μg/mL (n = 1) 1 μg/mL (n = 1)	0
*S.dysgalactiae equisimilis*	≤1 μg/mL (n = 2)	0
*streptococcus mitis*	≤1 μg/mL (n = 1)	0
*Enterococcus Faecium*	1 μg/mL (n = 1)	0

### 3.3 Evaluation of clinical effectiveness

Patients were followed up for at least 2 years after revision surgery. In the HC group, the average follow-up time was 28.57 months, whereas the LC group had a significantly longer follow-up time of 45.29 months (*P* < 0.05). Two patients (5.40%) expired in the HC group, one at 47 months post-discharge and the other at 15 months post-discharge. Both patients were died from heart failure. One patient (2.7%) in HC group was re-admitted for joint fusion due to recurrent infections.

Table 4 indicates that at admission, baseline WOMAC, SF-12, and HSS scores differed significantly between groups, with the HC group exhibiting poorer joint function and quality of life. This trend persisted at follow-up visits, although both groups demonstrated improvements in these scores. Specifically, the WOMAC score for the HC group was 18 (13.0, 18.0), significantly higher than the LC group at 7 (5.3, 8.8) (*P* = 0.001). The SF-12 score for the HC group was 33 (Thabit et al., 2019; He et al., 2021), lower than the 38 (37.75, 40) observed in the LC group (*P* = 0.001). The HSS score for the HC group was 73.0 (65.5, 83.5), while the LC group scored 85.0 (85.0, 90.0), showing a statistically significant difference (*P* = 0.038). The Harris scores for the high and low concentration groups were 83.0 (83.0, 84.0) and 87.0 (82.0, 91.0), respectively, with no statistically significant difference (P = 0.150).

**TABLE 4:** Comparison of WOMAC, SF-12, Harris, HSS scores and synovial WBC counts, PMN% in two groups.

Parameters	At admission				Follow-up/Upon discharge			
	HC group (n = 23)	LC group (n = 14)	P value	P value^a^	HC group (n = 23)	LC group (n = 14)	*P* value	P value^a^
WOMAC	69.0 (61.0, 74.0)	43.0 (38.5, 56.3)	0.001*	0.001*	18.0 (13.0, 18.0)	7 (5.3, 8.8)	0.001*	0.001*
SF-12	29.0 ± 4.82	34.0 ± 3.63	0.002*	0.087	33 (31, 33)	38 (37.8, 40)	0.001*	0.001*
Harris	44.0 ± 7.98	45.0 ± 2.81	0.774		83.0 (83.0, 84.0)	87.0 (82.0, 91.0)	0.150	
Hss	37.0 ± 3.59	49.0 ± 3.93	0.001*		73.0 (65.5, 83.5)	85.0 (85.0, 90.0)	0.038*	
Synovial WBC count (/μL)	45,050 (28,513, 66,016)	32,524 (32,524, 33,705)	0.026*	0.002*	5481^b^ (1,774, 5,481)	7106^b^ (5,474, 7,106)	0.009*	0.087
PMN %	94% (94%, 96%)	94% (94%, 94%)	0.300	0.721	63%^b^ (57%, 82%)	66%^b^ (66%, 67%)	0.359	0.003*

^a^ Inverse Probability of Treatment Weighting.

^b^ Upon discharge, *: significant difference, *P* < 0.05.

At discharge, inflammatory indicators were compared between the two groups. In the HC group, 14 patients (60.87%) had normal CRP levels (≤10 mg/L), compared to 11 patients (78.57%) in the LC group, with no significant difference (P = 0.307). Regarding normal ESR levels (≤30 mm/h), eight patients (34.78%) in the HC group had normal levels, versus six patients (42.86%) in the LC group, again showing no statistically significant difference (*P* = 0.732).

At discharge, serum WBC levels were normal in both groups, 5.64 (5.03, 6.47) vesus 5.19 (3.92, 6.50) for HC and LC group, respectively, without a statistically significant difference (*P* = 0.526). Additionally, the length of hospital stay did not differ significantly between the groups, with the HC group averaging 27.0 (19.0, 37.0) days compared to 20.5 (17.25, 26.25) days in the LC group (*P* = 0.109).

Before the initiation of vancomycin therapy, a significant difference was observed in the synovial WBC count between the two groups. The median count in the HC group was 45,050/μL (28,513, 66,016), which was higher than the 32,524/μL (32,524, 33,705) in the LC group (*P* = 0.026). At discharge, the synovial WBC count in the HC group decreased to 5,481/μL (1,774, 5,481), lower than the 7,106/μL (5,474, 7,106) observed in the LC group (*P* = 0.009), indicating a more pronounced reduction. However, no significant differences in synovial PMN (%) were noted between the HC and LC groups during hospitalization (Table 4).

Patients were grouped according to the site of PJI infection (knee joint and hip joint). At both admission and the end of follow-up, the HC patients exhibited relatively poorer joint function and quality of life compared with LC patients (HC patients with knee infection had worse WOMAC and HSS scores, while HC patients with hip infection had worse WOMAC and SF-12 scores). HC patients with both hip and knee infection had higher white blood cell counts at admission and showed a greater reduction at discharge compared with the LC patients (data not shown).

After inverse probability of treatment weighting (IPTW) adjustment, no statistically significant differences were observed between the two groups in the upon discharge of the synovial WBC count, the at administration of SF12 score and synovial PMN (%) (P > 0.05). Significant differences emerged in: WOMAC scores at admission and follow-up (P = 0.001), with the HC group showing higher than the LC group; follow-up SF-12 scores (P = 0.001), where the LC group was significantly higher; at-admission synovial WBC counts (P = 0.002), with higher in the HC group; PMN% upon discharge (P = 0.003), demonstrating lower in the HC group compared to the LC group.

### 3.4 Evaluation of drug safety

During hospitalization, DILI was noted in two patients (8.70%) in high concentration group, similar to that of the low group (1 patient, 7.14%) (*P* > 0.999). In the high concentration group, five patients (21.73%) experienced AKI, while only one patient (7.14%) in the low concentration group was reported; however, this difference was not statistically significant (*P* = 0.376). Regarding leukopenia, three patients (13.04%) in the high concentration group and four patients (28.57%) in the low concentration group were affected (*P* = 0.390). Both groups had three patients who required discontinuation of vancomycin therapy due to adverse drug reactions, representing 13.04% in the high concentration group and 21.43% in the low concentration group, with no significant difference (*P* = 0.653).

### 3.5 Prediction of vancomycin distribution in bone marrow and kidney using a PBPK model

**FIGURE 2:**
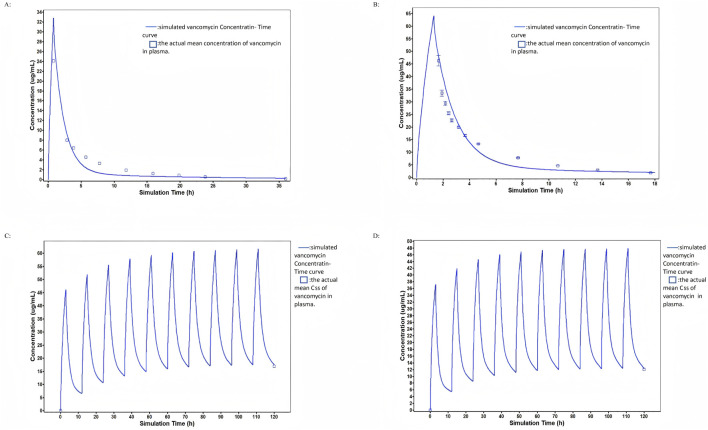
Model simulations of vancomycin concentration-time profiles from four studies in the training dataset: **(A)** Observed and PBPK model simulated vancomycin plasma concentrations after a single 1,000 mg intravenous dose in a young US population. **(B)** Observed and PBPK model simulated vancomycin plasma concentrations after a single 1,000 mg intravenous dose in a young Chinese population. **(C)** Observed and PBPK model simulated vancomycin plasma concentrations after multiple 1,000 mg doses every 12 h in the HC group. **(D)** Observed and PBPK model simulated vancomycin plasma concentrations after multiple 1,000 mg doses every 12 h in the LC group.

A PBPK model for intravenous vancomycin administration in U.S. population was first developed (Figure 2). The simulated PK profiles profiles closely matched the observed results from clinical trial data (Cutler et al., 1984), with AUC_0-t_ and C_max_ P/O ratio of 1.30 and 0.92, suggesting that the current assumptions of the vancomycin PBPK model are accurate. The model was then adapted to Chinese young adults. The plasma concentration-time profile predicted by the PBPK model for this population following intravenous vancomycin administration showed good agreement with the observed data, the AUC_0-t_ and C_max_ P/O ratio were 1.31 and 1.06 (Figure 2). Building on this PBPK model, physiological parameters were further adjusted to reflect the characteristics of HC and LC group in our study. The vancomycin concentration-time profiles in HC and LC group treated with multiple intravenous injections (1,000 mg per 12 h) were predicted. The Css P/O radio of HC and LC group were 0.99 and 1.04, respectively, indicating that the PBPK model was also able to accurately simulate the plasma concentration-time profiles of vancomycin under multiple dosing conditions, consistent with the treatment regimen used in our patient cohort (Figure 2).

**FIGURE 3:**
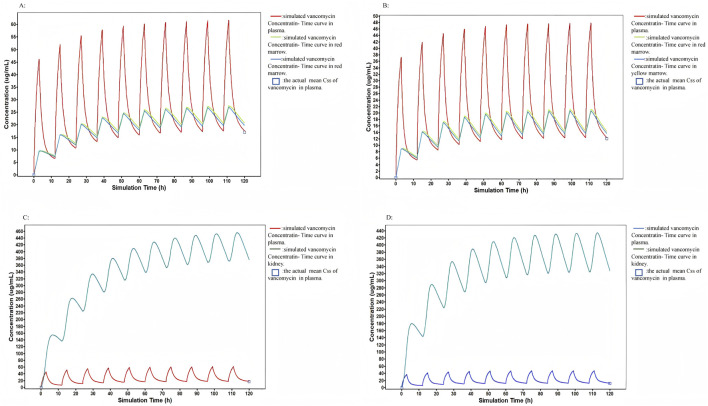
The model to predict vancomycin concentrations in the red marrow, yellow marrow and kidney: **(A)** Observed and PBPK model simulated concentrations in plasma, red marrow, and yellow marrow in the HC group. **(B)** Observed and PBPK model simulated concentrations in plasma, red marrow, and yellow marrow in the LC group. **(C)** Observed and PBPK model simulated concentrations in plasma and kidney in the HC group. **(D)** Observed and PBPK model simulated concentrations in plasma and kidney in the LC group.

We utilized the model to predict vancomycin concentrations in the red marrow, yellow marrow, and kidney (Figure 3). The results revealed that the HC group exhibited higher concentrations in both the bone marrow and kidney compared to the LC group. For red marrow, the vancomycin concentration in the HC group ranged from 20.66 to 27.34 μg/mL, while in the LC group, it ranged from 14.34 to 21.03 μg/mL. The concentration range in yellow marrow was similar to that of red marrow, with the HC group showing concentrations between 19.69 and 26.73 μg/mL, and the LC group between 13.58 and 20.47 μg/mL. In the kidney, the vancomycin concentration range for the HC and LC groups were 376.2–455.3 μg/mL and 327.7–432.9 μg/mL, respectively.

## 4 Discussion

To our knowledge, this is the first study to evaluate the effectiveness and safety of vancomycin therapy in PJI patients within two trough serum concentration ranges: low (10–15 μg/mL) and high (15–20 μg/mL). Our findings show that patients in the 15–20 μg/mL range demonstrated a greater reduction in synovial WBC count at discharge and a higher AUC/MIC value. Improvements in WOMAC, SF-12, or HSS scores were made, but no significance was noted as compared to those in the 10–15 μg/mL range. When analyzed separately by hip and knee joint cohorts, no statistically significant differences in synovial WBC counts were observed between admission and discharge (p > 0.05 for all comparisons). This absence of significance may be attributable to reduced statistical power resulting from diminished sample sizes in subgroup analyses. Following IPTW adjustment, certain parameters exhibited altered significance levels, but the overall trends remained consistent with and supportive of our primary conclusions. A comparative analysis of patients with different infection sites revealed no significant differences in final clinical outcomes between those with knee and hip joint infections, indicating that the anatomical location of infection has limited influence on treatment efficacy. Mortality and infection recurrence rates did not differ between the groups, nor did the incidence of adverse effects, including AKI, DILI, and leukopenia. Together, these findings suggest that while vancomycin Css levels of 10–20 μg/mL are generally effective and safe for PJI treatment, maintaining levels within 15–20 μg/mL may enhance infection control for for severe cases of PJI patients who present with higher synovial WBC account, without compromising patient’s safety, joint function or long-term quality of life.

Guidelines currently emphasize an AUC/MIC ratio of ≥400 as the key pharmacokinetic/pharmacodynamic (PK/PD) indicator of vancomycin effectiveness and recommend serum trough levels of 15–20 μg/mL as a surrogate marker for serious infections due to MRSA (22), the clinical benefits of maintaining higher vancomycin trough values in PJI cases have not been well documented. Moreover, Current vancomycin exposure effectiveness data originated largely from studies of bacteremia, pneumonia and infective endocarditis and none for PJI therapy. Treating PJI poses unique challenges due to the need for antibiotics to penetrate bone and synovial tissues, where biofilms with high antibiotic tolerance are common (Chapman et al., 2024). Pharmacokinetic studies have shown that vancomycin has limited penetration into bone and joint tissues, with a reported bone-to-plasma concentration ratio of 0.21–0.45 (Bue et al., 2018; Thabit et al., 2019; Graziani et al., 1988).

Some studies have explored methods to increase local vancomycin levels, such as direct intra-articular injection (He et al., 2021; Abuzaiter et al., 2023) and application of vancomycin powder at the surgical site (Ushirozako et al., 2021; Harper et al., 2023). However, the effectiveness and risks of these approaches remain debated, with intravenous route remaining the cornerstone of PJI treatment. Long-term vancomycin therapy, especially at higher trough concentrations, has been associated with risks such as nephrotoxicity and ototoxicity, particularly in elderly patients. Nevertheless, few studies have explored the impact of different vancomycin serum concentration ranges on PJI infection control, quality of life, and joint function over long-term follow-up. It remains unclear whether maintaining Css levels between 15 and 20 μg/mL, as opposed to 10–15 μg/mL, would confer additional benefits in terms of effectiveness and safety for PJI patients.

This study observed that, before initiating vancomycin treatment, patients in the high concentration group had a significantly elevated baseline synovial WBC count compared to those in the low concentration group. By discharge, however, the high concentration group demonstrated a notably lower synovial WBC count than the low concentration group, suggesting a more substantial reduction in inflammation. PBPK model revealed an approximately 20% higher vancomycin concentration in the bone marrow of the HC group compared to the LC group. Previous research has shown a strong and persistent upregulation of the proinflammatory environment in the joint-surrounding osseous scaffold in patients with PJI (Biedermann et al., 2023), suggesting that higher drug distribution to bone may contribute to more effective infection control. Maintaining vancomycin concentrations within the range of 15–20 μg/mL is associated with higher vancomycin levels in bone marrow, which may enhance inflammation resolution at the infection site and reduce the risk of infection recurrence. Based on these findings, maintaining a steady-state vancomycin concentration of 15–20 μg/mL may benefit patients with more severe PJI infections, particularly those with higher synovial WBC counts. This strategy could facilitate more rapid infection control, as defined by the Musculoskeletal Infection Society criteria.

A worse baseline renal function with a higher age was found with the HC group before initiation of vancomycin therapy, which may explain the increased drug exposure during the treatment. The age difference may also contribute to difference in baseline scores on the WOMAC, SF-12, and HSS assessments before vancomycin therapy. Notably, despite the renal functioning differences, the incidence of AKI did not differ between groups, indicating that vancomycin trough concentrations of 15–20 μg/mL remain relatively safe for elderly PJI patients with reduced renal function during hospital stay. The PBPK model predictions for PJI patients indicate that vancomycin concentrations in the kidneys differ by only 5%–15% between the HC and LC groups. This small difference may help explain the similar incidence of AKI observed between the two groups. Our findings are consistent with Yan’s study which demonstrated no increase in incidence of vancomycin induced AKI among a higher percentage of patients achieving a Css level of 15–20 μg/mL (Wei et al., 2023). This further supports the safety profile of vancomycin in this specific population.

Recently, AUC values (assuming a MIC_BMD_ of 1 mg/L) have been recommended to be maintained between 400 and 600 mg h/L to maximize effectiveness and minimize the likelihood of AKI. However, Casapao reported (Casapao et al., 2015) a significantly higher incidence of vancomycin treatment failure in patients with MRSA infective endocarditis when their AUC/MIC ratio was ≤600. And another systematic review (Aljefri et al., 2019) showed that AUC > 650 mg h/L was associated with a higher risk of nephrotoxicity. In this study, our findings showed when the AUC for vancomycin concentrations remained between 600 and 650 mg h/L, effective infection control was achieved in PJI patients without increasing the risk of AKI.

Follow-up results indicated that two patients in the high concentration group passed away. One patient, aged 98 at admission had concomitant disease including cerebrovascular disease, myocardial infarction, and pulmonary emphysema, reflecting generally poor health. The other patient, aged 81 at admission, had a history of atrial fibrillation. Both patients were elderly with chronic health conditions, and ultimately, both deaths were attributed to cardiovascular and cerebrovascular diseases.

This study had several limitations. First, this is a retrospective, single-center study with relatively small sample size which may limit result generalizability. Second, most patients in both groups were elderly with multiple comorbidities and on polypharmacy, complicating the assessment of other medications’ impact on AKI and DILI outcomes. Third, although there was no statistically significant difference in AKI incidence between the two groups, the high concentration group showed a trend toward a higher incidence of AKI compared to the low concentration group. Therefore, the impact of maintaining a higher concentration on kidney function warrants further investigation.

## 5 Conclusion

This study provides evidence that achieving a serum trough vancomycin concentration of 15–20 μg/mL may improve infection control for PJI patients who present with higher synovial WBC account, without adversely affecting safety, joint function, or long-term quality of life. Future research should focus on larger randomized controlled trials with extended follow-up periods to further clarify the effectiveness and safety of maintaining vancomycin Css levels between 15 and 20 μg/mL for PJI patients.

## Data Availability

The raw data supporting the conclusion of this article will be made available by the authors, without undue reservation.
